# Assessing Mental Health of Women Living in Karachi During the Covid-19 Pandemic

**DOI:** 10.3389/fgwh.2020.594970

**Published:** 2021-01-12

**Authors:** Shabnam Shamim Asim, Samrah Ghani, Maheen Ahmed, Anushae Asim, Afzal Fatima Karim Qureshi

**Affiliations:** ^1^Department of Gynecology and Obstetrics, Karachi Medical and Dental College, Karachi, Pakistan; ^2^Department of Internal Medicine, Dow University of Health Sciences, Karachi, Pakistan

**Keywords:** COVID-19, mental health, women, depression, anxiety, pandemic, Pakistan

## Abstract

**Introduction:** Women are more susceptible to mental health disorders and have been reported to experience higher levels of depression and anxiety during previous large-scale disease outbreaks. Stressful events like the COVID-19 pandemic can add extra burdens to women's already multifaceted lives. Keeping the gender implications of COVID-19 in mind can assist health care workers to offer more effective management. In our study, we aimed to assess the impact of COVID-19 on the mental health of women in Karachi, Pakistan and investigate the possible risk factors.

**Methods:** An online questionnaire was distributed to women on social media platforms in the month of June 2020. The questionnaire had two self-assessment scales, Patient Health Questionnaire (PHQ-9) scale which measures the symptoms of depression and General Anxiety Disorder (GAD-7) scale which measures anxiety.

**Results:** Three hundred and ninety three individuals completed the questionnaire with the mean age calculated to be 27.6 ± 11.7 years. Age, education, marital status, number of children, financial issues, employment status, smoking, comorbidities and mental illnesses were significantly associated with participants' mean anxiety and depression scores. The depression scores were generally higher compared to anxiety scores in each category. As the age increased, their scores decreased, with women aged 18–30 having a significantly higher mean depression and anxiety scores compared to women who were above 50. Severe anxiety was identified in 21.9% women and severe depression was noted in 17.8% women. A frightening number of 148 (37.7%) was found of women who had self-destructive thoughts at one time or another. Out of these women, surprisingly 97 (65.5%) individuals were not previously diagnosed with any mental illnesses.

**Discussion:** This study supports the existing literature regarding the disturbed psychological state of women close to the peak of the covid-19 pandemic. We noted increased percentage of depressive women as compared to studies conducted before the covid-19 era. This raises concern especially with our thought provoking finding of self-harm or suicidal thoughts among women. Most of our female population is also seen to be anxious. This study highlights the importance for help and support groups of mental health to effectively reach women during this period of social isolation.

## Introduction

The outbreak of pneumonia like cases of a novel etiology that was first observed in Wuhan, China, in December 2019 (1), swiftly spread across the globe and led to the pandemic now known as the coronavirus disease 2019 (COVID-19) (2). SARS-CoV-2, a member of severe acute respiratory syndrome-related coronavirus species, the virus that causes COVID-19, is predominantly transmitted by person-to-person contact via respiratory droplets (3).

In an attempt to curb its spread, countries all over the world have taken strict public health measures (4). Large-scale spread of COVID-19 has caused mass panic and anxiety (5), which are further amplified by introduction of lockdowns, travel restrictions and suspension of educational institutions (6). In Pakistan, the first case of COVID-19 was reported from Karachi on February 26, 2020 (7). As of August 13, 2020, there have been around 287,000 confirmed cases and more than 6,100 deaths in Pakistan. The province of Sindh has recorded the highest percentage of cases, with its capital city Karachi forming more than 30% of all cases in the country (8).

Historically, extensive outbreaks of infectious diseases have been linked to a variety of profound psychological effects among people (9, 10). In a study conducted in 2010 about public's response to influenza A H1N1 outbreak, it was found that 9.6 to 32.9% of people were “very worried” about contracting swine flu (9). Another study in Hong Kong concluded that 10 to 18% of participants appeared to have symptoms of depression, anxiety and post-traumatic stress disorder during the Severe Acute Respiratory Syndrome (SARS) epidemic (10). Factors that may affect the intensity of psychological impact among people include gender, age, income stability, place and mode of residence, presence of underlying chronic conditions, previous or existing psychiatric illness and presence of a relative diagnosed with or deceased due to the disease (11, 12).

Among these factors, it is notable that women are more prone to disorders of anxiety and depression (13) and have been reported to experience higher levels of anxiety during previous pandemics (14). Women also showed an anxiety risk that was 3.01 times higher than males in a study performed to assess the general publics' psychological health during the current COVID-19 pandemic in China (15). Disease outbreaks are known to multiply women's burdens with pre-existing stresses at work and home being enhanced as schools shut down and family members get infected (16–18). With Pakistan having a predominantly patriarchal society system, women are expected to experience higher degrees of unpaid care work, economic burden and domestic abuse in current periods of social isolation (19, 20).

Several studies were conducted to evaluate the mental health of high-risk groups like adolescents, students and health care workers during the COVID-19 pandemic in Pakistan (21–24). However, to the best of our knowledge after an extensive literature search, no studies were found to assess the psychological impact of this pandemic solely on women. Therefore, this study aims to determine the levels of depression and anxiety related to COVID-19 among adult women in Karachi, Pakistan and to identify potentially associated factors, to assist health care workers to provide a more effective and specific response to the affected women.

## Materials and Methods

This is a cross-sectional study designed to identify the effects of Covid-19 pandemic on the mental health of women in Karachi, Pakistan by an online questionnaire which was kept anonymous. A convenient sampling technique which engaged the general female population was used. Taking the anticipated frequency to be 50%, at a confidence interval of 95%, a sample size of minimum 384 was calculated. The inclusion criteria consist of all women living in Karachi who are above the age of 18, had access to internet and understood English, the language in which the questionnaire was composed. Females below the age of 18 were excluded from the analysis.

The quantitative data was collected on a validated online questionnaire which consists of informed consent, demographic data, Patient Health Questionnaire (PHQ-9) scale and General Anxiety Disorder (GAD-7) scale. The online questionnaire was distributed to women on different social media platforms through a google form link in the month of June 2020. The demographic data consists of general characteristics age, marital status, level of education, employment status, co-morbidities etc.

Previous studies on mental health during COVID-19 pandemic have widely used these two scales (25, 26), one of which was conducted on female health care workers in China (25). The PHQ-9 scale is used to measure symptoms of depression and has the total score range from 0–27 where 0–4 is minimal, 5–9 is mild, 10–14 is moderate, 15–19 is moderately severe and 20–27 is severe depression (27). The GAD-7 scale is used to measure anxiety and has the total score range from 0 to 21 where 0–4 is no anxiety, 5–9 is mild, 10–14 is moderate and 15–21 is severe anxiety (28).

Descriptive statistical analysis was carried out on a data set with IBM SPSS 23.0. All information gathered via google forms was recoded into variables. Missing values were coded as −1 so as not to affect results. Normality of data was tested using Shapiro-Wilk test. Both descriptive and inferential statistics involving Mann-Whitney U test and Kruskal Wallis H test were used to present results. For each test, a *P*-value <0.05 was considered statistically significant.

## Results

Out of 404 individuals who accepted to participate in the study, 393 completed the questionnaire, giving a response rate of 97%. The qualitative characteristics of the participants are summarized in Table 1. Of the 393 participants, 79.2% were aged 18–30, 11.8% were aged 30–50 and 9% were aged above 50. The mean age was calculated to be 27.6 years with a standard deviation of 11.7. One hundred and seven (27%) women were married, and of those women, 86 had children, with 51 women having 3–5 children. 66.8% women were undergraduates; 25.1% were post-graduates; 7.8% had received higher secondary education and 0.3% had received secondary education. More than half the participants (56.6%) reported to be students. 23.4% i.e., 92 women were employed, of whom 84 were working during the pandemic with 45 women working from home and 39 going to the workplace. The vast majority of participants (98.2%) were living with their families. Women living alone or with roommates were grouped together as the number was very small (7). 22.4% individuals suffered from comorbidities; 18.6% were previously diagnosed with mental illnesses; and 6.1% reported to be smoking. A relatively low proportion of women (15%) reported to exercise regularly for four times a week, while a high proportion of women (42.7%) did not exercise at all. Forty five percent women had relatives and friends diagnosed with COVID-19, while 6.1% women had relatives and friends who had died of COVID-19.

**Table 1:** Sociodemographic factors of participants.

**Characteristics**	***N* (%)**
**Age (years)**	27.6 ± 11.7
18–30	309 (79.2)
30–50	46 (11.8)
>50	35 (9.0)
**Marital status**	
Single	281 (72.4)
Married	107 (27.6)
**Have children**	
Yes	86 (22.2)
No	302 (77.8)
**Number of children**	
0–2	34 (40)
3–5	51 (60)
**Level of education**	
Secondary	1 (0.3)
Higher Secondary	30 (7.8)
Undergraduate	259 (66.8)
Post-graduate	97 (25.1)
**Work status**	
Student	219 (56.6)
Employed	92 (23.4)
Unemployed	76 (19.3)
**Working during the pandemic**	
Yes	84 (93.3)
No	6 (6.7)
**Workplace**	
Work from home	45 (53.6)
Going to workplace	39 (46.4)
**Living status**	
Living alone/roommates	7 (1.8)
Living with family	386 (98.2)
**Comorbidities**	
Yes	88 (22.4)
No	304 (77.6)
**Diagnosed with any mental illness**	
Yes	73 (18.6)
No	320 (81.4)
**Smoking**	
Yes	24 (6.1)
No	368 (93.6)
**Exercise**	
No	168 (42.7)
1–2 times a week	106 (27.0)
3–4 times a week	60 (15.3)
>4 times a week	59 (15.0)
**Currently faced any financial issues**	
Yes	96 (24.4)
No	297 (75.6)
**Acquaintances diagnosed with COVID**	
Relatives/friends diagnosed with COVID	177 (45)
Relatives/friends died from COVID	24 (6.1)
Both	20 (5.1)
Neither	172 (43.8)

The mean depression and anxiety scores related to participants' demographics are described in Table 2. The depression scores were generally higher compared to anxiety scores in each category. As the age increased, their scores decreased, with women aged 18–30 having a significantly higher mean depression score than women who were above 50 (*P* = 0.000). Single women had twice the depression score as married women, and the relation was seen to be statistically significant (*P* = 0.000). Similarly, there was a significant relation with women who had children and specifically 3–5 number of children with their depression scores being less compared to their counterparts (*P* = 0.000 and *P* = 0.018 respectively). The mean depression scores were similar with different levels of education, however, the relation between the two factors is statistically significant (*P* = 0.000). Students had a significantly higher depression score than employed and unemployed women (*P* = 0.000). Women who reported to be working from home had a higher depression score than those going to the workplace with the difference being statistically significant (*P* = 0.013). Individuals with chronic diseases had a significant association with their mean depression score (*P* = 0.014). Participants who faced financial problems during the pandemic and participants who reported to be smoking had significantly higher depression scores (*P* = 0.000 and *P* = 0.000, respectively). No significant difference in mean depression scores was observed among women who were working during the pandemic, women who lived with their families, women who exercised, and women who had a COVID+ patient among friends and relatives.

**Table 2:** Association of sociodemographic factors with mean depression and anxiety scores.

**Characteristics**	**Depression**	**Anxiety**
	**M ± SD**	**M ± SD**
**Age**		
18–30	12.9 ± 6.9	10.7 ± 5.9
30–50	5.7 ± 4.7	6.1 ± 5.1
>50	2.4 ± 2.8	3.2 ± 3.6
*P*-value	0.000^*^	0.000^*^
**Marital status**		
Single	13.2 ± 6.9	10.8 ± 5.9
Married	6.0 ± 5.8	6.1± 5.3
*P*-value	0.000^*^	0.000^*^
**Have children**		
Yes	4.7 ± 4.7	5.2 ± 4.6
No	13.1 ± 7.0	10.8 ± 6.0
*P*-value	0.000^*^	0.000^*^
**No of children**		
0–2	5.7 ± 4.4	6.7 ± 4.8
5-Mar	4.1 ± 4.5	4.1 ± 4.0
*P*-value	0.018^*^	0.005^*^
**Level of education**		
Secondary	20 ± 20	20 ± 20
Higher Secondary	13.3 ± 7.8	10.7 ± 6.3
Undergraduate	12.5 ± 7.1	10.5 ± 5.9
Postgraduate	6.9 ± 6.5	6.5 ± 5.7
*P*-value	0.000^*^	0.000^*^
**Work status**		
Student	12.9 ± 6.9	10.6 ± 6.1
Employed	7.8 ± 6.6	7.3 ± 5.8
Unemployed	10.3 ± 8.1	9.3 ± 6.2
*P*-value	0.000^*^	0.000^*^
**Working during the pandemic**		
Yes	8.1 ± 6.7	7.7 ± 5.8
No	3.3 ± 3.8	3.0 ± 3.5
*P*-value	0.063	0.043^*^
**Workplace**		
Work from home	9.7 ± 7.0	8.8 ± 5.8
Going to workplace	6.2 ± 5.9	6.5 ± 5.8
*P*-value	0.013^*^	0.054
**Living status**		
Living alone/roommates	6.2 ± 5.2	4.8 ± 3.4
Living with family	11.2 ± 7.4	9.4 ± 6.2
*P*-value	0.125	0.189
**Comorbidities**		
Yes	12.3 ± 8.5	10.9 ± 6.6
No	10.8 ± 7.1	8.9 ± 6.0
*P*-value	0.014^*^	0.103
**Diagnosed with any mental illness**		
Yes	16.2 ± 6.6	13.6 ± 5.3
No	9.9 ± 7.1	8.4 ± 6.0
*P*-value	0.000^*^	0.000^*^
**Smoking**		
Yes	16.8 ± 7.4	12.7 ± 6.0
No	10.7 ± 7.2	9.2 ± 6.2
*P*-value	0.000^*^	0.006^*^
**Exercise**		
No	11.4 ± 7.2	9.5 ± 6.4
1–2 times a week	11.5 ± 7.7	9.5 ± 6.1
3–4 times a week	11.5 ± 6.8	9.2 ± 5.6
>4 times a week	9.4 ± 7.9	8.9 ± 6.8
*P*-value	0.171	0.848
**Currently faced any financial issues**		
Yes	13.8 ± 7.6	11.6 ± 5.8
No	10.2 ± 7.1	8.6 ± 6.2
*P*-value	0.000^*^	0.000^*^
**Acquaintances diagnosed with COVID**		
Relatives/friends diagnosed with COVID	11.2 ± 7.2	9.4 ± 6.2
Relatives/friends died from COVID	11.6 ± 8.1	10.7 ± 5.8
Both	11.0 ± 8.0	7.7 ± 5.8
Neither	11.0 ± 7.5	9.4 ± 6.4
*P*-value	0.977	0.44

* *P-value < 0.05 is significant*.

Table 2 demonstrates that there is a statistically significant inverse relationship between age and mean anxiety scores with women aged 18–30 scoring thrice as high as women aged 50 above (*P* = 0.000). There is a significant relation between marital status and anxiety score with single women scoring higher than married women (*P* = 0.000). Women who did not have children had significantly twice as high anxiety scores as women who did have children (*P* = 0.000). While women who had 3–5 number of children had significantly lower scores than women who had 0–2 number of children (*P* = 0.005). Mean anxiety scores across different levels of education were similar but the relation was noted to be statistically significant (*P* = 000). Students had significantly higher mean anxiety scores compared to employed and unemployed women (*P* = 0.000). Among working women, the ones who were working during the pandemic had significantly higher anxiety scores than their counterparts (*P* = 0.043). A significant association was seen in individuals who reported to be smoking and their anxiety score (*P* = 0.006). Women who faced financial issues and women who had been diagnosed with mental illnesses had a significant relation with their anxiety scores (*P* = 0.000 and *P* = 0.000, respectively). There was no significant association of mean anxiety scores among women who worked from home, women who lived with their families, women with comorbidities, women who exercised, and those with a COVID+ patient among friends and relatives.

**Figure 1:**
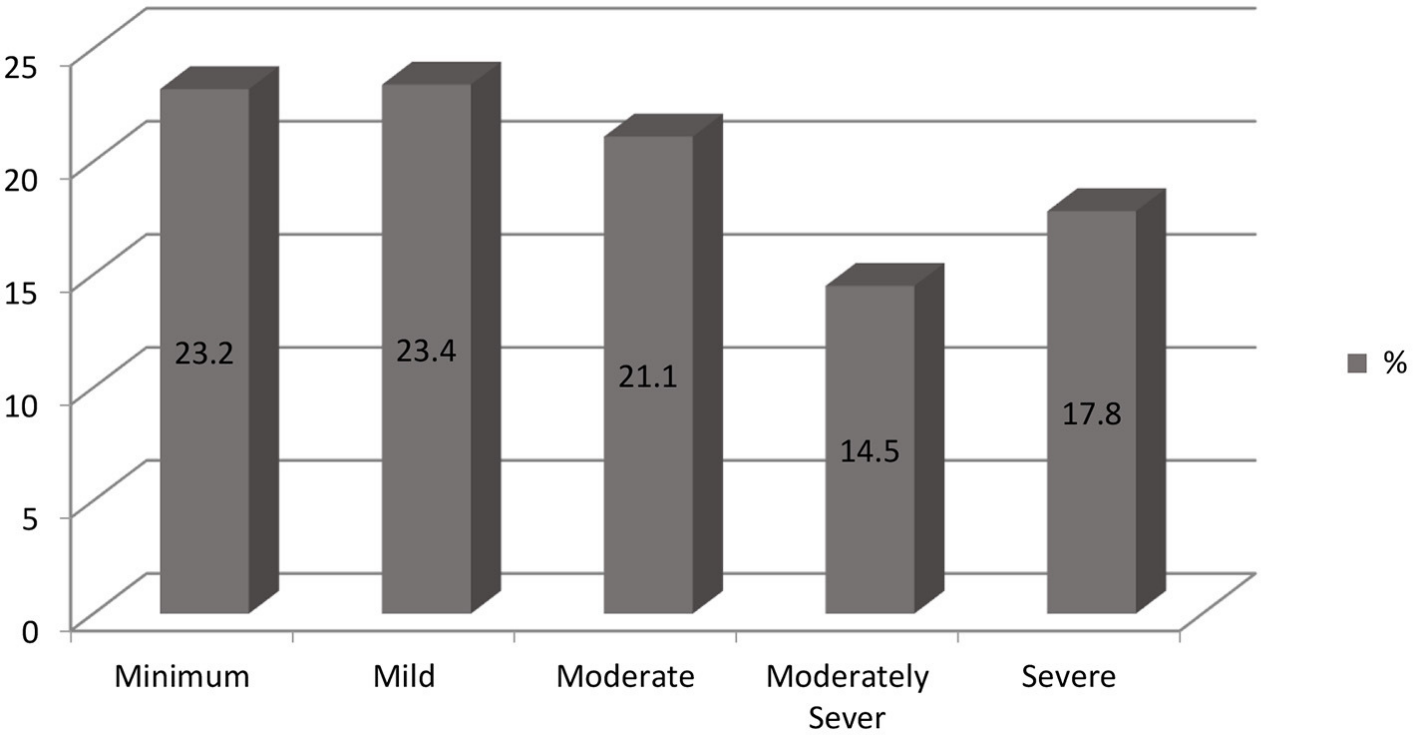
Distribution of participants PHQ-9 scale scores.

Figure 1 summarizes the depression scores of the participants. Although 18% participants were previously diagnosed with mental illnesses and 81% were not, all participants had notable anxiety and depression symptoms. All women showed degrees of depression with 23.2% suffering from minimum, 23.4% from mild, 21.1% from moderate, 14.5% from moderately severe and 17.8% from severe depression.

**Figure 2:**
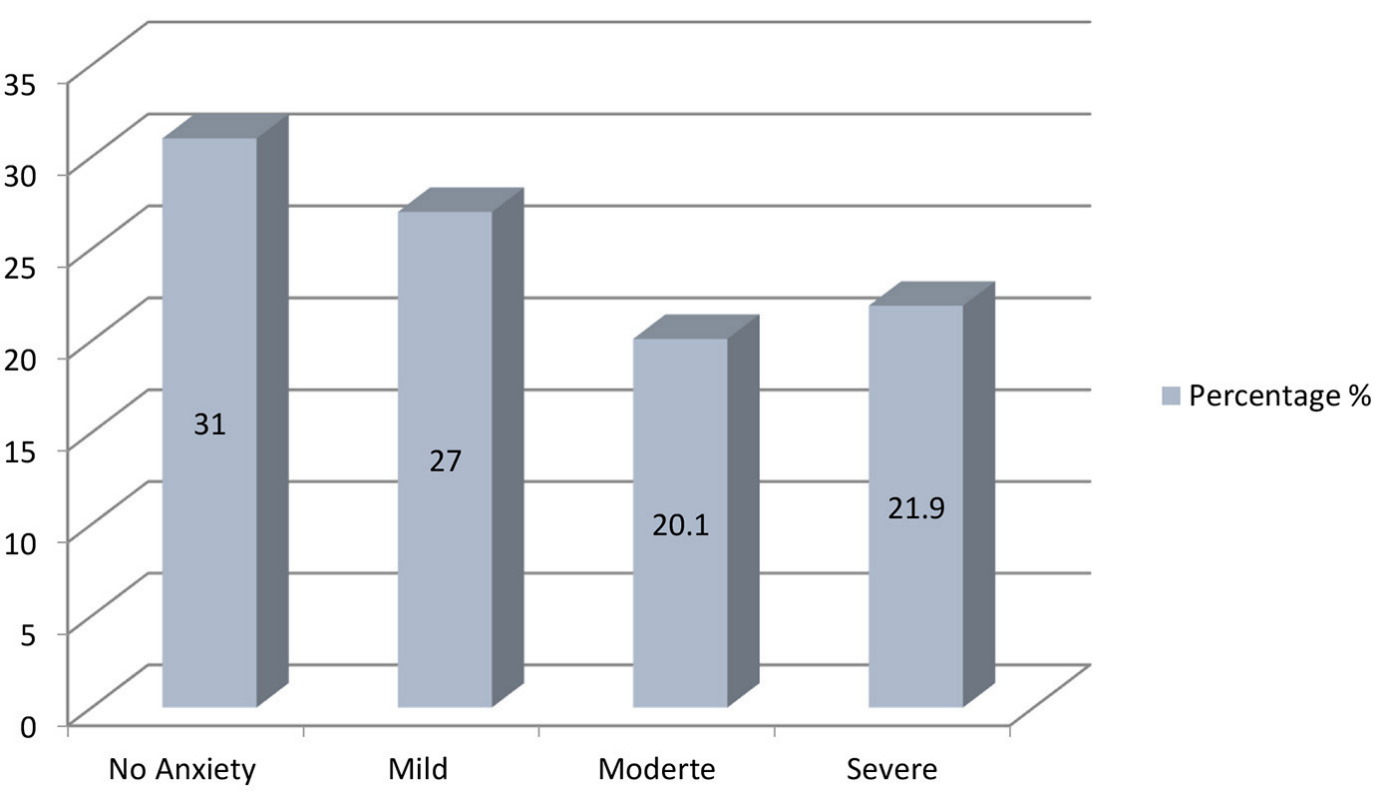
Distribution of participants GAD-7 scores.

Participants' anxiety levels are portrayed in Figure 2. There was roughly equal distribution of participants in all categories with 31% having no anxiety while 27% had mild, 20% had moderate and 21.9% had severe anxiety.

Table 3 depicts participants with thoughts of self-harm. The question 9 of PHQ-9 scale asked participants whether they had thoughts that they would be better off dead or hurting themselves. Women who picked the option of several days, more than half the days and nearly every single day were grouped together as “yes.” An alarming number of 148 (37.7%) was found of women who had self-destructive thoughts at one time or another. Out of these women, surprisingly 97 i.e., 65.5% individuals were not previously diagnosed with any mental illnesses.

**Table 3:** Participants with thoughts of self-harm.

**Parameter**	**Total**	**Previously diagnosed with mental illness**	
	***N* (%)**	**Yes**	**No**
**Thoughts of self-harm**			
Yes	148 (37.7)	51 (34.5)	97 (65.5)
No	245 (62.3)	22 (9.0)	223 (91.0)

## Discussion

This study acknowledges COVID related increase in depression and anxiety levels among adult women living in the cosmopolitan city, Karachi. It has been evidenced through research that women are prone to developing mental health problems (29–32). This study was conducted close to the dates the pandemic was estimated to reach its peak in Pakistan (33), hence it was hypothesized that there might be alarming levels of depressive and generalized anxiety disorders among the chosen sample. It was also later observed that the country witnessed its highest number of cases during our data collection period, in the month of June (34).

In a study assessing psychological distress, inclusive of depression and anxiety, Qiu et al. (35) report higher scores among the young adult group. This finding is supported by our research where higher scores for depression and anxiety are seen among women aged 18 to 30 as well. This pattern can be explained with findings of Cheng et al. (36) that young people have access to overwhelming information through social media which may be increasing their psychological burden. Association of age is found to be significant with depression in our study, which is supported by a study conducted in USA (37). However, it is mostly seen to be in contrast with other studies (11, 15, 26, 31). Similarly, age and anxiety are found to be significantly related in our study which is consistent with findings reported by a nationwide survey conducted in Italy (31). Nevertheless, it is in contrast with a few other studies too (11, 15). These variances might be due to the differences in the context and sample population.

An unanticipated finding in our study shows that women with no children have significantly higher levels of depression and anxiety both whereas women with children only report mild levels of depression and anxiety. This is in accordance with findings reported by a study in Italy where childlessness was associated with depression (31). This could be due to lack of loneliness and a sense of fulfillment associated with having children in south-Asian communities in particular.

It is expected that students, in any outbreak of an infectious disease, might suffer from various psychological burdens as it may be a direct impediment to their ongoing education, with number of corona virus patients rising, all educational institutes were shut down nationwide. A study in China states students to be dealing with high levels of depression and anxiety (29). Odriozola-González et al. (38) in their study conducted in a university report significantly higher levels of depression and anxiety among students when compared to university employees.These findings are supported by our study, where students seem to have suffered a higher degree of mental impact when compared to employed or unemployed people.

Depression and occupation or being an employee are seen to be significantly associated in a few studies while no such association is seen between these variables and anxiety (15, 31). In total contrast to these findings, our study shows working during the pandemic to be significantly associated with anxiety and not depression. In a study in Turkey no significant relationship is seen between working during the pandemic and anxiety or depression (11). Such differences however can be expected in different geographical regions.

Our study shows people having comorbidities with a significantly higher mean score for depression, while no significant association is seen with mean anxiety scores which is in accordance with the study conducted in Turkey (11). A study in Italy however shows both anxiety and depression to be significantly associated with history of medical problems (31). These findings could be accredited to COVID-19's worse progression with various chronic illnesses (39).

Smoking, in our study, has been shown to be significantly associated with higher mean values for depression and anxiety both, which could be attributed to the adverse progression and severe outcomes associated with the sars-cov-2, if contracted (40).

It was anticipated that having people affected with the contagious virus in an individual's close vicinity might have had a direct mental impact on the individual. However, to our surprise our study found no significant relationship existed between having friends, relatives or acquaintances with covid-19 and depression or anxiety scores. The finding is in contrast with some existing literature (15, 26). A research conducted in turkey also reports significantly higher means scores for depression and anxiety for the same variable (11). The disparity could be attributed to contextual differences.

A study discussing the psychological burden in women during the Severe Acute Respiratory Syndrome (SARS) epidemic in Hong Kong showed 28.6% women to have mild depression or higher, which is a higher number than noted before the pandemic (41). Similarly, to assess the occurrence of depression before and during the pandemic, we compare our results with studies conducted among women living in Karachi before the spread of this contagious virus. These studies state overall depression among women to be a little <40% (42, 43). Another study reports prevalence of depression and anxiety among women living in Karachi to be 30% (44). Gadit and Mugford (45) also report frequency of depression in Karachi to be 35.7%, however, the percentage is inclusive of both genders. On the other hand, our study shows over 53.4% of women with major depression, calculated taking a PHQ-9 score of 10 and above. This was based on the findings of Kroenke et al. (27) who reported a PHQ-9 score ≥10 to have a sensitivity of 88% and a specificity of 88% for major depression. The increased percentage of depressive women seen in this study as compared to studies conducted before the covid-19 era, reaffirms our assumption that the pandemic may have had a direct psychological effect on women. The study on the SARS epidemic shows a statistically significant relationship between all age groups and depression (41). Our study supports this existing literature. These similar findings reflect a pattern which is expected in any outbreak of an infectious disease; however, it must be noted that our study is inclusive of younger age groups as well.

Huang and Zhao (46) in their study reported 34.1% of females to have anxiety symptoms using the GAD-7, taking score 9 or higher as presence of anxiety, whereas our study, using the same scale, shows 42% women to have moderate anxiety levels or higher i.e., score 10 and above. The percentages both the studies show seem close. The minimal difference noted might be explained with differences in understanding of the same scale. Khan et al. (47) in their study conducted prior to the COVID-19 pandemic have reported, taking the GAD-7 score 5 and above to show some degree of anxiety, 45.5% of anxious women. Using the same score as a threshold in an attempt to draw a comparison, we find our study reports 69% of women to have anxiety, which is a much higher number than noted before the pandemic. It can be concluded from the available data that the pandemic may have had a direct effect on the anxiety levels found in our population. It must be noted that the relationship between COVID-19 and depression, or anxiety could not be founded conclusively in our study; however, it can be strongly inferred as a possible cause. A key adverse effect of the pandemic has been said to be loneliness and increased social isolation (48) which have been linked with anxiety and depression strongly in other studies (49, 50), therefore this is an issue which warrants immediate attention.

A thought provoking finding in our study is the vast number of people considering self-harm or suicide. As shown in Table 3, while 62.3% of the people never thought of hurting themselves or being dead, 37.7% of the studied population had thoughts relating to self-harm, ranging from several days to nearly every day which calls for immediate action to help. A study in UK shows 17.9% of women having similar thoughts, however the percentage is much smaller than ours (51). In a paper discussing suicides in Pakistan, we found that there have been sixteen suicidality related cases since January 2020 which were all associated with the COVID-19 pandemic, of which two of the stated cases were women reportedly killing themselves because of suspected infection and economic distress (52). This also brings our attention toward our results where 24.4% of the women studied faced financial issues, which is a smaller percentage compared to 40% of women reportedly affected during the SARS epidemic (41) however, still holds importance.

Our limitations include use of a small sample size. More precise results can be obtained with a similar survey conducted on a larger scale. Due to convenience sampling technique, there was an oversampling of a certain group i.e., students and to avoid that, the next research may divide the population in groups and various sets. Given that this study is a cross-sectional survey, it at best serves as a snapshot of the situation. It cannot be made sure through our study that the psychological impact was due to the pandemic specifically, as life events or any personal factors were not adjusted for. To interpret whether COVID affected the prevalence of depression and anxiety, we have compared our results with studies conducted in our population prior to the pandemic, however it must be noted that some comparisons were drawn between different assessment tools for the same disorders. The study included self-assessment questionnaires and no professional diagnosis was made for any of the mentioned ailments above. The study does not conclusively establish a relationship between COVID and depression, or anxiety since cofounders were not accounted for. Ethics approval from a Human Research Ethics Committee was not obtained due to implementation of a strict lockdown. However, the questionnaire circulated online comprised of validated scales and had an elaborate consent form included. Anonymity of the collected data was maintained to ensure that any information cannot be traced back to the participant. All participants voluntarily consented to take part in our study and there was no in-person or physical human recruitment.

To the best of our knowledge, this is the first study that has been conducted in Pakistan exclusively targeting the mental health of women during the pandemic, so filling a gap in the literature. Our study also highlights the need for help regarding mental health to immediately reach women during this period of social isolation. Women make an asset to this country and directly affect lives of other people here and availability of treatment options for their mental health is of paramount importance at the moment.

The sample chosen does not reflect the entire population of Pakistan and future researches can be conducted at a national level in this area. A validated questionnaire could be created accounting for personal factors to precisely assess depressive disorders and anxiety before and during the pandemic. Our research aimed at women living in an urban city, and there's no knowledge available on psychological health of women making up the rural population of this country and therefore any future researches involving them may prove beneficial. We recommend studies to be conducted to see how available and in reach are the psychological help options for women in this country during this pandemic, or otherwise.

## Conclusion

This research shows the mental health of women in Karachi to be noticeably affected during the pandemic with an alarming finding of thoughts regarding self-harm. Younger females in our context were seen to be more vulnerable. Students due to a sudden break in their on-going education seem to be dealing with more depressive and anxious thoughts. Women suffering with chronic illnesses have had a higher mental impact than healthier women. Keeping in view these findings, essential assistance should be made available through online support groups, awareness though television or social media and telemedicine. Moreover, informative messages through short message services or call services may help reduce the overall public panic, and therefore help reducing the anxiety levels found in our population.

## Data Availability Statement

The original contributions presented in the study are included in the article/Supplementary Material, further inquiries can be directed to the corresponding author/s.

## Author Contributions

SA supervised the research and acted as the mentor. SG ran all the data analysis and statistical analysis. AA, MA, AQ, and SG drafted various sections of the manuscript. All authors contributed to the article and approved the submitted version.

## Conflict of Interest

The authors declare that the research was conducted in the absence of any commercial or financial relationships that could be construed as a potential conflict of interest.
